# Development of GPCR Modulation of GABAergic Transmission in Chicken Nucleus Laminaris Neurons

**DOI:** 10.1371/journal.pone.0035831

**Published:** 2012-04-24

**Authors:** Zheng-Quan Tang, Yong Lu

**Affiliations:** Department of Anatomy and Neurobiology, Northeast Ohio Medical University, College of Medicine, Rootstown, Ohio, United States of America; Hotchkiss Brain Institute, University of Calgary, Canada

## Abstract

Neurons in the nucleus laminaris (NL) of birds act as coincidence detectors and encode interaural time difference to localize the sound source in the azimuth plane. GABAergic transmission in a number of CNS nuclei including the NL is subject to a dual modulation by presynaptic GABA_B_ receptors (GABA_B_Rs) and metabotropic glutamate receptors (mGluRs). Here, using in vitro whole-cell patch clamp recordings from acute brain slices of the chick, we characterized the following important but unknown properties pertaining to such a dual modulation: (1) emergence of functional GABA synapses in NL neurons; (2) the temporal onset of neuromodulation mediated by GABA_B_Rs and mGluRs; and (3) the physiological conditions under which GABA_B_Rs and mGluRs are activated by endogenous transmitters. We found that (1) GABA_A_R-mediated synaptic responses were observed in about half of the neurons at embryonic day 11 (E11); (2) GABA_B_R-mediated modulation of the GABAergic transmission was detectable at E11, whereas the modulation by mGluRs did not emerge until E15; and (3) endogenous activity of GABA_B_Rs was induced by both low- (5 or 10 Hz) and high-frequency (200 Hz) stimulation of the GABAergic pathway, whereas endogenous activity of mGluRs was induced by high- (200 Hz) but not low-frequency (5 or 10 Hz) stimulation of the glutamatergic pathway. Furthermore, the endogenous activity of mGluRs was mediated by group II but not group III members. Therefore, autoreceptor-mediated modulation of GABAergic transmission emerges at the same time when the GABA synapses become functional. Heteroreceptor-mediated modulation appears at a later time and is receptor type dependent in vitro.

## Introduction

Synaptic transmission is dynamically modulated by G-protein-coupled receptors (GPCRs) acting as autoreceptors or heteroreceptors [1], [2]. Commonly found among these receptors are metabotropic glutamate receptors (mGluRs) [3], [4], [5] and type-B GABA receptors (GABA_B_Rs) [6], [7], which are activated by the two most prevalent excitatory and inhibitory neurotransmitters in the vertebrate CNS, glutamate and GABA, respectively. These receptors play important modulatory roles in a variety of auditory nuclei by mediating long-term plasticity, regulating transmitter release, and altering neuronal response properties [8], [9], [10]. A dual modulation of GABA release by both presynaptic GABA_B_Rs and mGluRs has been found in a number of CNS nuclei including two avian auditory nuclei involved in coding of temporal information of sounds [11], [12], [13]. In such cases, GABA_B_Rs function as autoreceptors modulating GABA release via a use-dependent feedback mechanism, whereas mGluRs function as heteroreceptors modulating GABA release. Because these previous studies have been mainly focused on characterizing the neuromodulation in relatively mature tissues, a number of important questions pertaining to the development of such a dual modulation remain unanswered. Does the autoreceptor-mediated modulation emerge at the same time when the GABA synapses start functioning through postsynaptic ionotropic receptors? Which one appears first, the modulation mediated by autoreceptors or the modulation mediated by heteroreceptors? Are the physiological conditions, under which the heteroreceptors are activated by endogenous glutamate and exert their modulatory effects, similar to those for autoreceptors?

The avian nucleus laminaris (NL) circuit constitutes an excellent model system to address these questions. Both the anatomy and the physiological function of the NL have been well characterized [14], [15]. NL neurons receive both glutamatergic and GABAergic inputs, providing the sources for the two native neurotransmitters that activate mGluRs and GABA_B_Rs involved in the dual modulation of GABA release. The development of the glutamatergic input to the NL, which originates from bushy cells in the cochlear nucleus magnocellularis (NM), has been well established. Synaptic connections between NM and NL form at E8/9 when these two nuclei originating from the auditory anlage start to be structurally separated [16], [17]. Physiological recordings in brain slices have demonstrated that these synapses become functional (defined as the appearance of synaptic responses mediated by postsynaptic ionotropic receptors evoked by activating their afferent fibers) at E10/11, a few days after synapse formation [18], [19]. In contrast, the development of the GABAergic input to the NL, which originates primarily from the superior olivary nucleus (SON), is not fully understood. While anatomical data have shown that GABA terminals to the NL have little presence at E9–11 and a few GABAergic fibers are present at E12–14 [20], physiological data about the onset of functional GABA synapses are lacking. Therefore one of the goals of this study was to determine when the GABA synapses in NL became functional.

Regarding the temporal onset of GABA_B_R- and mGluR-mediated modulation of GABA release, two intriguing and intuitive hypotheses can be formed. First, modulation mediated by the autoreceptors starts functioning prior to that by heteroreceptors. Even before synaptogenesis, both GABA_B_Rs and GABA_A_Rs are expressed on neuronal membranes and the GABA signaling via these receptors participates in many cellular events in early development such as cell growth, survival, migration, and synaptogenesis [21]. The presence of both GABA_B_Rs and GABA_A_Rs in early development renders the possibility of autoreceptor-mediated modulation to appear immediately after the synapses become functional. Therefore, we predicted that GABA_B_R-mediated modulation took place prior to mGluR-mediated modulation of GABAergic transmission in the NL. The second hypothesis predicted that the physiological conditions that induced the endogenous activity of GABA_B_Rs in neuromodulation differed from those for mGluRs. Multiple factors, such as the spiking activity level of the presynaptic terminals, the spatial-temporal features of the transmitter diffusion, clearance mechanisms of transmitters, receptor affinity, and subcellular location of the receptors, may be involved in determining the extent of activation of these receptors [22], [23]. Being present on the presynaptic terminal membranes surrounding the synaptic cleft or sometimes located directly in the cleft area, autoreceptors are physically close to the transmitter release sites, enhancing the chance for activation of the autoreceptors by spilled over transmitter molecules. In contrast, heteroreceptors are generally located farther in distance from the release sites of their endogenous transmitter, and the chance of direct synapsing onto the synaptic terminals where these heteroreceptors are located is rare [22], [23]. Therefore, modulation mediated by endogenous activity of heteroreceptors may require more intense synaptic activity of the corresponding inputs than that mediated by autoreceptors.

## Methods

### Slice preparation and in vitro whole-cell recordings

Fertilized chicken eggs were purchased from Meyers Hatchery. Eggs were incubated using an RX2 Auto Turner and a Clearview Brooder (Lyon Electric Co., Chula Vista, CA). Brainstem slices (250–300 µm in thickness) were prepared from chicken embryos, as described previously [24], with modification of the components of the artificial cerebrospinal fluid (ACSF) used for dissecting and cutting the brain tissue. The modified ACSF, which is a glycerol-based solution [25], contained (in mM): 250 glycerol, 3 KCl, 1.2 KH_2_PO_4_, 20 NaHCO_3_, 3 HEPES, 1.2 CaCl_2_, 5 MgCl_2_, and 10 dextrose, pH 7.4 when gassed with 95% O_2_ and 5% CO_2_. The procedures were approved by the Institutional Animal Care and Use Committee (IACUC) at Northeast Ohio Medical University, and are in accordance with NIH policies on animal use. Slices were incubated at 34–36°C for 1 hr in normal ACSF containing (in mM): 130 NaCl, 26 NaHCO_3_, 3 KCl, 3 CaCl_2_, 1 MgCl_2_, 1.25 NaH_2_PO_4_ and 10 dextrose. ACSF was constantly gassed with 95% O_2_ and 5% CO_2_ (pH 7.4). For recording, slices were transferred to a 0.5 ml chamber mounted on a Zeiss Axioskop 2 FS Plus microscope (Zeiss, Germany) with a 40×- water-immersion objective and infrared, differential interference contrast optics. The chamber was continuously superfused with ACSF (2–2.5 ml/min) by gravity. The microscope was positioned on the top center of an Isolator CleanTop II and housed inside a Type II Faraday cage (Technical Manufacturing Corporation, Peabody, MA). Recordings were performed at 34–36°C, controlled by a Single Channel Temperature Controller TC324B (Warner Instruments, Hamden, CT).

Patch pipettes were drawn on an Electrode Puller PP-830 (Narishige, Japan) to 1–2 µm tip diameter using borosilicate glass Micropipets (inner diameter of 0.86 mm, outer diameter of 1.60 mm) (VWR Scientific, Seattle, WA). The electrodes had resistances between 3 and 7 MΩ when filled with a solution containing (in mm): 105 K-gluconate, 35 KCl, 5 EGTA, 10 HEPES, 1 MgCl_2_, 4 ATP-Mg, and 0.3 GTP-Na, with pH of 7.2 (adjusted with KOH) and osmolarity between 280 and 290 mOsm. The Cl^−^ concentration (37 mM) in the internal solution approximated the physiological Cl^−^ concentration in NL neurons, measured previously [13]. Placement of the recording electrodes was controlled by a motorized micromanipulator MP-225 (Sutter Instrument, Novato, CA). The liquid junction potential was 10 mV, calculated using a software package by Barry [26], and data were corrected accordingly.

The voltage clamp experiments were performed with an AxoPatch 200B amplifier (Molecular Devices, Union City, CA). Data were low-pass filtered at 3–10 kHz, and digitized using a Data Acquisition Interface ITC-18 (Instrutech, Great Neck, NY) at 20 kHz. Recording protocols were written and run using the acquisition and analysis software AxoGraph X (AxoGraph Scientific, Australia).

All chemicals and drugs were obtained from Sigma (St Louis, MO) except for (±)-1-Aminocyclopentane-*trans*-1,3-dicarboxylic acid (tACPD), 3-[[(3,4-Dichlorophenyl)methyl]amino]propyl] diethoxymethyl)phosphinic acid (CGP52432), (RS)-α-Cyclopropyl-4-phosphonophenylglycine (CPPG), (2S)-2-Amino-2-[(1S,2S)-2-carboxycycloprop-1-yl]-3-(xanth-9-yl) propanoic acid (LY341495), 1,2,5,6-Tetrahydro-1-[2-[[(diphenylmethylene)amino]oxy]ethyl]-3-pyridinecarboxylic acid hydrochloride (NNC 711), DL-*threo*-β-Benzyloxyaspartic acid (DL-TBOA), and (3S)-3-[[3-[[4-(Trifluoromethyl)benzoyl]amino]phenyl]methoxy]-L-aspartic acid (TFB-TBOA), which were obtained from Tocris (Ballwin, MO). All drugs were bath-applied except for 5-Aminomethyl-3-hydroxyisoxazole (muscimol), which was applied with pressure ejection (puff application) by using a multi-channel picospritzer (General Valve, Fairfield, NJ). Muscimol (10 µM) was prepared in ACSF containing 6,7-Dinitroquinoxaline-2,3-dione (DNQX, 50 µM) and D-(-)-2-Amino-5-phosphonopentanoic acid (APV, 100 µM), antagonists for ionotropic glutamate receptors (AMPA and NMDA receptors, respectively). Puff electrodes were prepared using the same pulling methods as producing recording electrodes except that the puff electrodes had larger tip diameter (2–5 µm). The puff electrode was placed above and lateral to the recoded cell at a distance of 50–100 µm. Positive pressure (30–70 kPa, duration of 200 ms) was used to eject the muscimol-containing solution.

### Synaptic stimulation and recordings of synaptic responses

Extracellular synaptic stimulation was performed using concentric bipolar electrodes with a tip core diameter of 127 µm (World Precision Instruments, Sarasota, FL). Because of the small size of NL and limited number of slices that can be obtained from young embryos (e.g., 1–2 slices at E11–13 that contain distinguishable NL) [18], we did not classify cells based on frequency regions. In relatively older embryos (>E15), we intentionally recorded cells from approximately the mid/high-frequency regions in order to avoid complications in our interpretation introduced by tonotopic distribution of neuronal properties. Neurons in the NL receive GABAergic inhibitory inputs primarily from the ipsilateral SON [27], [28], [29]. To activate the GABAergic pathway, the stimulation electrode was placed using a Micromanipulator NMN-25 (Narishige, Japan) in the area immediately lateral to the NL where the ipsilateral SON fibers travel to innervate the NL. To activate both the GABAergic and the glutamatergic pathways, the stimulation electrode was placed in an area dorsal and lateral to the NL, where fibers from the ipsilateral NM and SON fibers are mixed. Single or train stimulations at different frequencies (pulse duration of 200 µs) were delivered through a Stimulator A320RC (World Precision Instruments, Sarasota, FL). Optimal stimulus parameters were selected for each cell to give rise to reliable postsynaptic currents. Before each synaptic stimulation protocol was applied, a 5 mV hyperpolarizing command (duration of 5 ms) was given to monitor series resistance and input resistance during the experiment. Cells with >20% changes in their series resistance during the recordings were discarded.

Evoked inhibitory postsynaptic currents (IPSCs) were recorded in the presence of DNQX (50 µM) and APV (100 µM). The synaptic stimulation was repeated 6–12 times under each experimental condition. The raw traces were averaged off-line and the peak values of IPSCs were measured. The average of the peak values of the IPSCs was considered as one data point, representing the averaged IPSC under the experimental condition. These methods have been established in our previous studies [11], [12].

Graphs were constructed in Igor (Wavemetrics, Lake Oswego, OR). Means and standard errors of the mean (SEM) are reported (n in parenthesis indicates number of cells). ANOVA post hoc Fisher's test was used for statistical analyses, and p<0.05 was considered statistically significant.

## Results

### Onset of GABA_A_R responses in NL neurons

**Figure 1:**
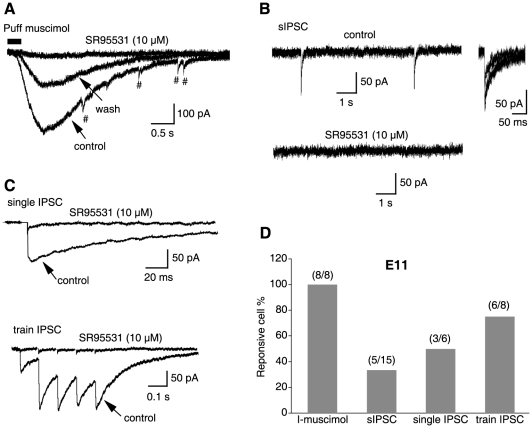
GABAergic transmission starts functioning in about half of the NL neurons at E11. ***A***, Puff application of a selective GABA_A_R agonist muscimol (10 µM) evoked an inward current that was nearly completely blocked by SR95531 (10 µM), an antagonist specific for GABA_A_Rs, indicating the presence of functional GABA_A_Rs on NL cell membrane. Spontaneous IPSCs (sIPSCs) are indicated by the symbol #. ***B***, Voltage clamp recordings from a sample cell showing sparsely distributed sIPSCs that were eliminated by SR95531, indicating that GABA_A_Rs mediated the sIPSCs. Shown on the right are superimposed sIPSCs at an enlarged time scale. ***C***, Electrical shocks (upper panel: single pulse stimulation; lower panel: train stimulation at 10 Hz) delivered to the GABAergic afferents to the NL evoked inward currents sensitive to SR95531, indicating the presence of functional GABA synapses. Stimulus artifacts are blanked for clarity. ***D***, Percent of responsive cells under different recording conditions. All cells showed responses to muscimol, 5 out of 15 cells expressed sIPSCs, 3 out of 6 cells responded to the single pulse stimulation, and 6 out of 8 cells responded to the train stimulation. Cells were voltage clamped at −60 mV. DNQX (50 µM) and APV (100 µM), AMPAR and NMDAR blockers, respectively, were present in all experiments.

Because neuromodulation mediated by GABA_B_Rs activated by endogenous GABA relies on the presence of functional GABA synapses, we first characterized the onset of GABAergic transmission in the NL. Based on the anatomical data showing that GABA terminals to the NL have little presence at E9–11 and a few GABAergic fibers are present at E12–14 [20], we chose E11 as the earliest age to study the onset of physiological responses mediated by GABA_A_Rs in NL neuron. After obtaining whole-cell voltage clamp recordings from NL neurons, we used a series of stimulating approaches combined with pharmacological agents to evoke whole-cell responses sensitive to GABA_A_R blockers (Fig. 1). Ionotropic glutamate receptor blockers (50 µM DNQX and 100 µM APV) were present in all experiments. Puff application of a selective GABA_A_R agonist muscimol (10 µM), which was used to bypass the presynaptic GABA terminals and activate postsynaptic receptors directly, evoked inward currents (termed I-muscimol) in all cells studied. The inward currents were nearly completely blocked by 6-Imino-3-(4-methoxyphenyl)-1(6*H*)-pyridazinebutanoic acid (SR95531, 10 µM), an antagonist specific for GABA_A_Rs (Fig. 1A, D), indicating the presence of functional postsynaptic GABA_A_Rs on NL cell membrane at E11. Some spontaneous IPSCs (sIPSCs) were readily observed in the sample cell (Fig. 1A). This observation was further confirmed by prolonged recordings, in which 5 out of 15 cells showed sparsely distributed sIPSCs within a five-minute recording window. The sIPSCs were eliminated by SR95531, indicating that GABA_A_Rs mediated the currents (Fig. 1B, D) and GABA synapses started functioning in at least some NL cells at E11. Electrical shocks delivered to the presumably GABAergic afferents to the NL also evoked inward currents sensitive to SR95531 (Fig. 1C), further confirming the presence of functional GABAergic synapses. Cells were more reliably responsive to train stimulation (10 Hz 5 pulses) (6 out of 8 cells) than single-pulse stimulation (3 out of 6 cells) (Fig. 1D), possibly caused by facilitation of transmitter release under the train stimulation.

### Modulation of GABAergic transmission mediated by GABA_B_Rs emerges prior to that by mGluRs

**Figure 2:**
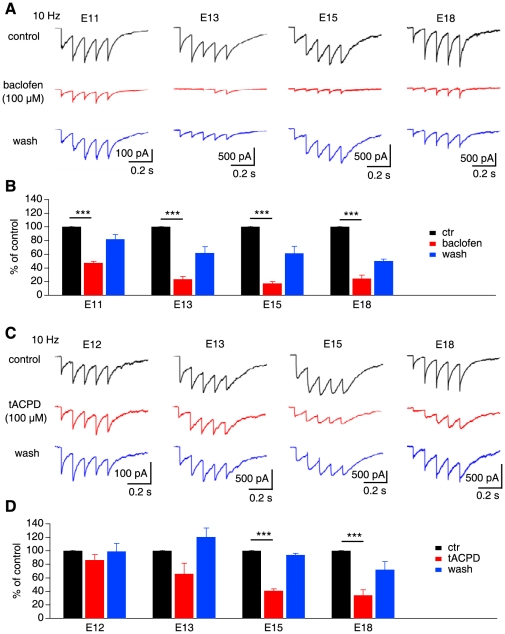
Modulation of GABAergic transmission in NL neurons by autoreceptors emerges prior to that by heteroreceptors. ***A***, Effects of GABA_B_R agonist baclofen (100 µM) on IPSCs of NL neurons obtained from chicken embryos ages of E11, E13, E15, and E18. Inhibition of the IPSCs by baclofen was observed in all cells. ***B***, Summary data showing that baclofen (100 µM) reduced IPSCs significantly in all ages tested (n = 6, 7, 7, and 5 for E11, E13, E15, and E18, respectively). ***C***, Effects of mGluR agonist tACPD (100 µM) on IPSCs of NL neurons obtained from chicken embryos ages of E12, E13, E15, and E18. ***D***, Significant suppression of IPSCs by tACPD (100 µM) was detected in E15 and E18 but not at E12 or E13 (n = 6, 5, 8, and 7 for E12, E13, E15, and E18, respectively). In this and subsequent figures, bars represent means ± SEM. NS: not significant (p>0.05), *p<0.05, **p<0.01, and ***p<0.001 (ANOVA post hoc Fisher's test).

Modulation of GABA release in NL neurons by autoreceptors (GABA_B_Rs) starts prior to that by heteroreceptors (mGluRs). This conclusion is based on the experiments in which we studied the effects of respective agonists for GABA_B_Rs and mGluRs on IPSCs of NL neurons obtained from animals of different ages. We elicited IPSCs with a low frequency (5 or 10 Hz, 5 pulses) train stimulation, and then a potent GABA_B_R agonist baclofen at its saturating concentration (100 µM) was applied. Significant suppression of the IPSCs was observed at the earliest age we studied (E11), and the suppression became stronger at E13, and remained strong in later ages (Fig. 2A, B; Table 1; n = 6, 7, 7, and 5 cells for E11, E13, E15, and E18, respectively).

**Table 1:** Summary of numerical data on the effects of baclofen (100 µM) and tACPD (100 µM) on the amplitude (pA) of IPSCs of NL neurons obtained at different ages.

age (n)	control	drug	washout
	baclofen		
E11 (6)	−191.3±28.9	−91.0±14.9** *	−148.0±18.2
E13 (7)	−269.4±72.2	−51.0±10.2***	−150.1±34.5
E15 (7)	−547.0±84.8	−89.0±15.8***	−419.3±77.9
E18 (5)	−526.4±162.8	−107.8±32.6***	−329.0±80.5

	tACPD		
E12 (6)	−171.9±38.5	−137.8±27.2	−160.7±38.5
E13 (5)	−260.4±64.4	−156.0±53.0	−321.5±103.6
E15 (8)	−517.3±73.4	−195.1±26.1***	−291.8±60.4
E18 (7)	−730.7±208.4	−168.0±32.2***	−473.0±51.7

Means ±1 SEM are shown. n: number of cells.

* p<0.05,

** p<0.01, and

*** p<0.001 (ANOVA post hoc Fisher's test).

To date, there are 8 members of mGluRs identified, and they are further divided into 3 groups (group I: mGluR1 and 5; II: mGluR2 and 3; and III: mGluR4, 6, 7, and 8) based on their homology, pharmacology, and signal transduction pathways [3], [4], [5]. To determine the temporal onset of mGluR-mediated modulation of GABA release in NL, we studied the effects of tACPD (100 µM), an agonist that can activate non-selectively most members of mGluRs [3], on IPSCs of NL neurons obtained at different ages. In contrast to the early onset of baclofen effects, significant suppression of IPSCs by tACPD (100 µM) was detected in E15 and E18 but not in earlier embryos (E12 or E13) (Fig. 2C, D; Table 1; n = 6, 5, 8, and 7 cells for E12, E13, E15, and E18, respectively). Because modulatory effects of tACPD were not observed at E12/13, studies using animals of earlier ages were unnecessary. It is noted that the time course of the IPSCs was different among the sampled cells. This is possibly due to the combined effects of two factors, one being developmental changes and the other being tonotopic specializations of neuronal properties. Supporting the effects of the second factor, we recently found that the time course of the postsynaptic GABA_A_ currents in NL neurons differed between different characteristic frequency (CF) regions. Compared to low-CF (LF) neurons, middle/high-CF (MF/HF) neurons had significantly slower IPSCs. To account for these distinct GABA_A_ responses, we showed that MF/HF neurons exhibited more prominent asynchronous release of GABA (our unpublished observations).

### Endogenous activity of GABA_B_Rs is stimulus frequency dependent

**Figure 3:**
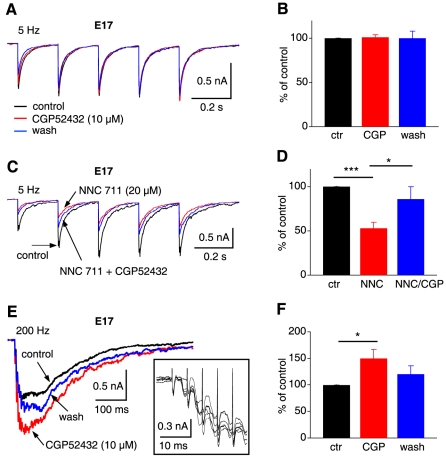
Endogenous activity of GABA_B_Rs is stimulus frequency dependent. ***A,*** Average IPSC traces of one NL neuron (E16–17) in response to train stimulations at 5 Hz (5 pulses) under the conditions of control, GABA_B_R antagonist CGP52432 (10 µM), and washout. ***B,*** IPSC peak amplitude normalized to the control showed that CGP52432 did not have significant effects on the IPSCs elicited at 5 Hz (n = 7), indicating lack of endogenous GABA_B_R activity under this stimulation condition. ***C & D***, Blocking GABA uptake by NNC 711 (20 µM) reduced IPSCs elicited at 5 Hz. In the presence of NNC 711, CGP52432 (10 µM) increased IPSC amplitude significantly (n = 7), revealing endogenous GABA_B_R activity. ***E & F***, In response to blockade of GABA_B_Rs by CGP52432 (10 µM), a significant increase in IPSC amplitude was observed at the stimulus frequency of 200 Hz (n = 8). The inset in panel E shows six superimposed individual IPSCs obtained under control conditions, at enlarged scales. Only the responses to the first five stimulus pulses (without blanking the stimulus artifacts) are shown to indicate low noise levels of the recordings.

Because higher input frequencies are expected to trigger strong and long-lasting release of GABA and likely subsequent activation of presynaptic GABA_B_Rs via transmitter spillover, we predicted that the level of endogenous GABA_B_R activity was stimulus frequency dependent. To test this hypothesis, we elicited IPSCs in NL neurons (E16–17) using train stimulations at low (5 or 10 Hz) and high (200 Hz) frequencies, and examined the effects on the IPSCs of a GABA_B_R antagonist CGP52432. At the concentration used (10 µM), CGP52432 is supposed to achieve saturating block of GABA_B_Rs [30]. In response to low frequency stimulation (5 or 10 Hz, 5 pulses), IPSC amplitude was unchanged when CGP52432 (10 µM) was applied (Fig. 3A, B; control: −479.4±89.7 pA; CGP52432: −468.0±79.5 pA, washout: −498.2±102.6 pA, n = 7, p>0.05), indicating lack of GABA_B_R activity induced by endogenous GABA under this stimulus condition. To enhance the chance of observing endogenous GABA_B_R activity on IPSCs elicited at low stimulating frequencies, we blocked the uptake of GABA, expecting that the transmitter molecules may have longer life span in the synaptic cleft and surrounding areas, facilitating the activation of presynaptic GABA_B_Rs. Inhibition of GABA uptake by NNC 711 (20 µM) reduced IPSCs elicited at 5 or 10 Hz (control: −746.3±119.9 pA; NNC 711: −364.0±56.7 pA, n = 7, p<0.001), suggesting the possibility of endogenous activity of GABA_B_Rs in depressing GABA release. Such effects induced by blocking uptake mechanisms have been observed at glutamate synapses [31]. In the presence of NNC 711, blockade of GABA_B_Rs by CGP52432 (10 µM) increased IPSC amplitude significantly (Fig. 3C, D; NNC 711: −364.0±56.7 pA; NNC 711 plus CGP52432: −599.4±116.6 pA, n = 7, p<0.05), revealing endogenous GABA_B_R activity. When tested at a high stimulating frequency of 200 Hz, which approximates the sound-evoked discharge rates of SON neurons *in vivo* [32], NL neurons generated temporally summated IPSCs with a long decay time course. CGP52432 (10 µM) alone (without blocking GABA uptake) significantly increased the normalized IPSC amplitude by 49.7±16.8% (Fig. 3E, F; control: −1171.6±374.9 pA; CGP52432: −1529.8±445.0 pA, washout: −855.6±179.2 pA, n = 8, p<0.05), indicating activation of the autoreceptors by synaptically released GABA under physiologically relevant stimulations. Along with our previous data showing that CGP52432 significantly increased the IPSCs evoked at 100 Hz [13], these results indicate that activation of presynaptic GABA_B_Rs is stimulus frequency dependent, and presynaptic GABA_B_Rs are activated in response to increased GABA release at high stimulation frequencies, forming a feedback mechanism controlling the GABAergic strength in NL neurons.

### Endogenous activity of mGluRs is stimulus frequency dependent and receptor specific

**Figure 4:**
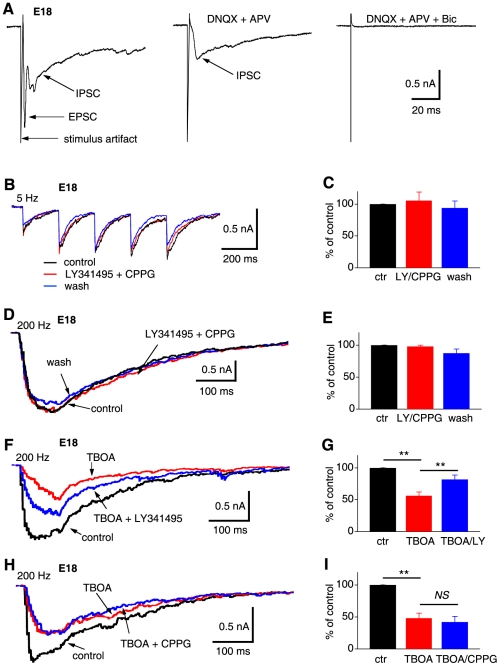
Endogenous activity of mGluRs is stimulus frequency dependent and receptor specific. ***A***, Concurrent activation of glutamatergic and GABAergic pathways to NL neurons (E15–19). Cells used in this figure responded to the synaptic stimulation with both a fast EPSC and a slow IPSC. DNQX and APV eliminated the EPSC, and a specific GABA_A_R antagonist bicuculline (40 µM) eliminated the IPSC. ***B & C*** **,** Antagonists able to block all mGluRs (4 µM LY341495 plus 10 µM CPPG) did not have significant effects on the IPSCs elicited at 5 or 10 Hz (n = 5), indicating lack of endogenous mGluR activity under low frequency stimulation conditions. ***D & E*** **,** Further increasing the stimulating frequency to 200 Hz still failed to induce endogenous mGluR activity (n = 5). ***F & G***, Endogenous activity of mGluRs was observed under the condition of high frequency (200 Hz) stimulation combined with inhibition of glutamate uptake. Furthermore, such endogenous activity was mGluR type specific. Inhibition of glutamate uptake by TBOA (DL-TBOA 50 µM plus TFB-TBOA 10 µM) reduced IPSCs elicited at 200 Hz. In the presence of TBOA, blockade of group II mGluRs by low concentration of LY341495 (10 nM) increased IPSC amplitude significantly (n = 5). ***H & I***, In contrast, in the presence of TBOA, blockade of group III mGluRs by CPPG (5 nM) had no effects on IPSC (n = 5).

Although exogenous agonists of mGluRs induced inhibition of GABA release, the physiological significance of such effects can be questioned if endogenous activity of mGluRs cannot be detected [33]. However, when present, endogenous activity of heteroreceptors (mGluRs in this case) may be induced under different stimulation conditions from those of autoreceptors. In order to study the activity of mGluRs activated by synaptically released glutamate, we placed the stimulation electrode in an area dorsal and lateral to the NL to activate both the GABAergic and glutamatergic pathways. Concurrent activation of GABAergic and glutamatergic pathways to NL neurons (E15–19) was observed in response to the same single stimulus (Fig. 4A). Then we examined the effects of mGluR antagonists under different stimulation conditions.

Antagonists at concentrations that are able to block all mGluRs (4 µM LY341495 plus 10 µM CPPG) [4], [5] did not have significant effects on the IPSCs elicited at 5 or 10 Hz (Fig. 4B, C, control: −383.6±74.6 pA; LY341495 plus CPPG: −409.6±85.9 pA, washout: −361.0±76.9 pA, n = 5, p>0.05), indicating lack of endogenous mGluR activity under low frequency stimulus conditions. Increasing the stimulating frequency to 200 Hz, which approximates the discharge rates of NM neurons *in vivo* [34], [35], still failed to induce any significant endogenous mGluR activity (Fig. 4D, E, control: −1118.4±359.7 pA; LY341495 plus CPPG: −1117.8±367.8 pA, washout: −910.8±279.6 pA, n = 5, p>0.05). These results suggest highly efficient clearance mechanisms for released glutamate at the synapses in NL, even though high-rate synaptic activity may delay the clearance process [36]. In order to enhance the chance of detecting endogenous mGluR activity, we blocked glutamate uptake systems, expecting to increase glutamate accumulation in the synaptic cleft and induce activation of mGluRs on GABAergic terminals by glutamate spillover [31], [37], [38]. Endogenous activity of mGluRs was indeed observed under the condition of high frequency stimulation (200 Hz) combined with full inhibition of glutamate uptake by perfusion of DL-TBOA (50 µM) and TFB-TBOA (10 µM) (Fig. 4F, G), both of which are glutamate transporter blockers, with the later being more glial specific. Furthermore, such endogenous activity was mGluR type specific. Inhibition of glutamate uptake by TBOA (DL-TBOA 50 µM plus TFB-TBOA 10 µM) significantly reduced IPSC elicited at 200 Hz (Fig. 4G, control: −1063.0±327.2 pA; TBOA: −567.2±193.3 pA, n = 5, p<0.01; Fig. 4I, control: −705.4±100.6 pA; TBOA: −360.8±94.0 pA, n = 5, p<0.01). In the presence of TBOA, specific blockade of group II mGluRs by low concentration of LY341495 (10 nM) [5] increased IPSC significantly (TBOA: −567.2±193.3 pA; TBOA plus LY341495: −819.8±243.7 pA, n = 5, p<0.01). In contrast, in the presence of TBOA, specific blockade of group III mGluRs by low concentration of CPPG (5 nM) [5], [39] had no effects on IPSCs (Fig. 4 H, I, TBOA: −360.8±94.0 pA; TBOA plus CPPG: −318.2±96.0 pA, n = 5, p>0.05).

## Discussion

We report that: 1) the GABAergic transmission in NL neurons starts functioning at E11; 2) the modulation of GABAergic transmission by autoreceptors takes place prior to that by heteroreceptors; and 3) endogenous activity of GABA_B_Rs and mGluRs is stimulus frequency dependent, and the endogenous activity of mGluRs is group specific.

The emergence of functional GABA synapses shows a short time delay in relative to that of glutamate synapses in NL neurons. Postsynaptic ionotropic glutamate receptors [19] and postsynaptic GABA_A_Rs (present study) were detectable in all cells at the earliest ages studied (E9 and E11, respectively). The early appearance of ionotropic glutamate and GABA_A_ receptors is not surprising because postsynaptic receptor-mediated signaling exists well before presynaptic terminals arrive to innervate developing neurons [21]. Soon after formation of glutamate synapses, synaptic responses emerge. At E10, evoked EPSCs mediated by both AMPARs and NMDARs are observed in about 75% cells [19], and evoked EPSPs are present in all cells studied at E11 [18], [19]. In contrast, synaptic GABA_A_R activity, either in the form of spontaneous or stimulus-evoked IPSCs, was detected in only about half of the neurons at E11, indicating that the development of the GABAergic transmission has a roughly 1-day delay relative to the development of the glutamatergic transmission. We cannot exclude the possibility that functional GABA synapses might appear in some cells at earlier ages, however, based on the present study, the proportion of cells that express functional GABA synapses is expected to be much lower than that at E11. Although the functional GABA synapses emerged with a time delay compared to glutamate synapses, the GABA_B_R-mediated modulation of GABA release took place a few days earlier than mGluR-mediated modulation (E11 vs. E15). The later appearance of heteroreceptor-mediated modulation may correlate with the functional expression of mGluRs, the time of hearing onset, and/or the development of the glutamatergic synapses. Although all three groups of mGluRs are expressed in NL at E13 (our unpublished observation), signaling transduction pathways linking mGluRs to the regulation mechanisms of GABA release may not be present at E13 and earlier ages. The emergence of mGluR activity on regulating GABA release at E15 may reflect the establishment of such signaling pathways, likely driven by the fast maturation of the glutamatergic inputs during the period of development after hearing onset, which occurs at about E11/12 [40]. The increase in synaptic activity mediated by glutamate, especially the patterned spontaneous spiking activity between E13–18 in NM and NL neurons [41], may facilitate expression of membrane mGluRs and establishment of their signaling transduction pathways.

GABA_B_Rs and mGluRs belong to the same subfamily of GPCRs, and share many structural and functional similarities including signaling transduction pathways [1]–[7]. While it awaits to be demonstrated whether these two different types of receptors co-localize on the presynaptic terminals of the GABA synapses in NL neurons, and whether they exert their modulatory actions independently and/or synergistically, we can make some implications based on our results of percent inhibitions of IPSCs induced by the respective agonists of GABA_B_Rs and mGluRs at different ages (Fig. 2). At E11–12, baclofen caused a significant inhibition (53%) of IPSCs while tACPD caused a statistically non-significant inhibition (13%). The total inhibition (66%) could imply two independent pathways for these two different types of receptors, if the total inhibition continued to be less than 100% in older ages. However, at E13, E15 and E18, the total inhibition increased to 111%, 142% and 142%, respectively, exceeding the predicted value (about 100%) if the two pathways were completely independent. These results suggest that GABA_B_Rs and mGluRs at the GABA synapses of NL neurons may share at least some signaling transduction pathways in modulation of GABA release.

Endogenous activity of both GABA_B_R- and mGluR-mediated modulation of GABAergic transmission expressed stimulus frequency dependence. However, the stimulus conditions under which such endogenous activity was detected differed. Specifically, the requirements to activate autoreceptors seemed to be less robust than those to activate heteroreceptors. Endogenous GABA_B_R activity at low stimulus frequencies was detected when GABA uptake was blocked. In contrast, endogenous mGluR activity was not elicited by low frequency stimulations of the glutamatergic pathway, even in the presence of glutamate uptake blockers. At more physiological relevant stimulus frequencies such as 100 Hz [13] or 200 Hz (present study), endogenous GABA_B_R activity was detectable even without blocking GABA uptake. In contrast, endogenous mGluR activity was not detected at high frequencies of 100 Hz [13] or 200 Hz (present study), unless glutamate uptake was blocked. The relative ease of activation of the autoreceptors has been observed at glutamate synapses as well [42], [43].

To be physiologically relevant, the regulation of GABA release by endogenous mGluRs must rely on glutamate spillover from glutamatergic terminals located nearby the GABA synapses. Since glutamate spillover was first discovered in 1997 in the hippocampus [44], it is commonly found in numerous CNS nuclei [45], [46], [47]. However, because of the strong uptake mechanisms of glutamate into glial cells [22], spillover of glutamate may only occur when a burst of spike activity is present, especially in vitro [45]. Presumably, the appearance of endogenous activity of mGluRs as heteroreceptors modulating GABA release in the NL may be facilitated by the morphological development of the dendrites in NL. In early developmental stages (E8/9), NL dendrites are of about equal length across different characteristic frequency (CF) regions. After a tremendous growth in dendritic arborization combined with apoptosis of NL cells during development, the dendrites in middle/high-CF regions shrink while those in low-CF regions grow in length, forming a gradient in dendritic length along the tonotopic axis [48], [49], a process influenced by astrocyte-secreted factors [50]. In addition, the inhibitory inputs to NL neurons are distributed on the soma as well as along the dendrites, where the glutamatergic synapses are located [51], [52]. These morphological changes could bring the two synaptic inputs closer in space, enhancing the chance of activation of mGluRs located on presynaptic terminals of the GABA synapses, and rendering heteroreceptor-mediated modulation of GABAergic transmission possible. In our present study, we intentionally recorded cells from slices obtained in older embryos (>E15) in approximately the mid/high-frequency regions in order to avoid complications in our interpretation introduced by tonotopic distribution of neuronal properties. It remains interesting for future studies to examine whether NL neurons from high CF regions display stronger endogenous mGluR activity than neurons from low CF regions. Such a notion can be supported by the observation that endogenous activity of mGluRs in regulating GABA release in NM neurons is readily detected under low frequency (10 Hz) stimulation without blocking glutamate uptake [12]. The ease of inducing mGluR activity in NM neurons is possibly due to the fact that NM neurons receive morphologically huge [53] and physiologically powerful [54] glutamatergic inputs (Endbulb of Held synapses) onto their cell bodies, where the inhibitory synapses are also located [55].

Finally, both groups II and III mGluRs suppress GABA release in NL via presynaptic mechanisms, and endogenous activity of mGluRs was demonstrated when antagonists able to block all mGluRs were applied [13]. The present study extended these previous findings by revealing that the endogenous activity was group specific in that group II antagonist LY341495 but not group III antagonist CPPG produced significant increases in IPSCs in the presence of glutamate uptake blockers (Fig. 4). Different signaling pathways involved in these two groups of mGluRs in modulating neurotransmission may account partly for this observation. Both group II and III mGluRs can regulate transmitter release via affecting presynaptic voltage-gated calcium channels [3], [4], [5]. However, group III mGluRs may cause presynaptic modulation of GABA release evoked only by neuronal activity, while additional mechanisms for the modulation mediated by group II mGluRs could exist [13], increasing the chance of detecting endogenous activity of group II mGluRs. Both groups of receptors have high affinity for glutamate, with group II receptors being more sensitive than group III receptors [3], [56]. We speculate that under in vivo conditions, if activation of both groups is possible, group II mGluRs may be activated under low-frequency activity, exerting a tonic modulation of GABA release and the activation of group III receptors may require more intense activity of glutamate synapses. Once being activated, these mGluRs work in synergy to prevent saturation of heteroreceptor-mediated regulation of GABA release in NL, ensuring proper synaptic strength of the GABAergic input and its modulatory function on ITD coding.
